# Integration of ICTs in teaching practices: propositions to the SAMR model

**DOI:** 10.1007/s11423-022-10169-x

**Published:** 2022-12-08

**Authors:** Rute Nogueira de Morais Bicalho, César Coll, Anna Engel, Maria Cláudia Santos Lopes de Oliveira

**Affiliations:** 1 Department of Distance Education in the Federal Institute of Brasília, Brasília, Brazil; 2 Department of Cognition, Development and Educational Psychology at the Faculty of Psychology, University of Barcelona, Barcelona, Spain; 3 Department of Cognition, Development and Educational Psychology at the Faculty of Psychology, University of Barcelona, Barcelona, Spain; 4 Educational and Developmental Psychology at the Institute of Psychology, University of Brasília, Brasília, Brazil

**Keywords:** Teaching practices, New learning ecology, Integration of ICTs, SAMR model, COVID-19 pandemic

## Abstract

This article aims to analyze the experiences of teachers on the uses of ICTs in the development of teaching practices within the framework of a new learning ecology. We use the SAMR model to scale the levels of ICTs contributions in each practice. 116 teachers from a public educational institution in Brazil answered a questionnaire during the pandemic scenario, while conducting emergency remote teaching. Teachers declared to develop the teaching practices with the integration of ICTs at the levels of Augmentation and Modification by correspondence to the SAMR model. Based on the analyzes carried out, we propose some reflections that help to rethink this model and to understand the second-order barriers that prevent the effective integration of ICT in teachers’ practices.

## Introduction

Digital Information and Communication Technologies (ICT) play a significant role today, favoring changes in different sectors of human activities: industries, services, professional fields, etc. In the educational context, many countries started to invest in technologies, promoting its importance in master plans and school curricula (Law et al., 2011). Currently, ICTs gain new meanings due to the challenges of emergency remote teaching during the COVID-19 pandemic.

The insertion of ICTs in the educational scenario has become a topic of pedagogical and scientific discussion since the end of the twentieth century. However, this debate has intensified in the current century, as a result of programs aimed at the digital transformation of school systems, which emerged in some countries as an effect of policies to stimulate innovation and promote global economic competitiveness (Lyddon 2019; McKnight et al., 2016). For a long time, the concept that the insertion of ICTs in schools could reshape the teaching and learning processes prevailed, and its insertion was rationalized above all in terms of positive impacts, considered a catalyst for educational reforms demanded in official government documents (Blundell et al., 2020).

It soon became evident that insertion, or mere access to technology, does not necessarily result in its integration into teaching practices, as its uses may continue to reflect traditional pedagogies (Coll & Engel, 2022; Coll et al., 2008a; Prestridge 2017). Certainly, access to technology favors the breaking of external barriers, called first-order barriers, as it does not depend directly on the actions of teachers. However, the effective integration of technologies implies overcoming second-order barriers, constituted especially by the beliefs and attitudes of teachers that affect their experiences in relation to the uses of ICTs (Backfisch, et al., 2021; Blundell et al., 2020; Geer et al., 2015; Kimmons & Hall, 2018; Prestridge, 2017; Seufert et al., 2021; Tondeur, et al., 2016; Vongkulluksna et al., 2018).

The effective integration of ICTs into teaching practices reveals a complex process, as their uses are modulated by multiple factors that influence the development of educational practices (Backfisch, et al., 2021; Seufert et al., 2021). In this sense, several models, structures and theories were developed in an effort to guide managers, researchers and teachers in the complex task of integrating technologies into the pedagogical routine (Backfisch, et al., 2021; Baz et al., 2018; Blundell et al., 2020; Flanagan 2016; Seufert et al., 2021). Among the models, we can mention the most recurrent in the literature, known by the acronyms: TPACK, SAMR, TIM e RAT.

In this article, we emphasize the SAMR model for its growing popularity among teachers (Baz et al., 2018; Hilton 2015; Kimmons 2018). Specifically, our objective is to analyze teachers' experiences on the declared uses of ICTs and understand how they integrate these technologies into the development of their teaching practices by reference to the levels indicated in the SAMR model. Our research questions are: 1. What are the potentials and limitations of the SAMR model to understand the process of integrating ICT in teaching practices? 2. How can the data from this survey improve the SAMR model? 3. How can the data from this research help to understand the integration of ICT in teaching practices with a view to overcoming second-order barriers?

From a sociocultural perspective, we understand ICTs as cultural artifacts and, as such, objects of meaning capable of mediating the teaching and learning process when they are used in different ways by the teachers. Understood in this way, the uses of ICTs allow teachers to self-regulate their practice, with the potential to introduce important cognitive, affective and psychological changes related to the educational process (Coll & Engel 2022; Coll & Martí 2014; Coll et al., 2008a; 2008b).

Bearing this perspective in mind, in the next topic we will present the SAMR model, highlighting its potential and limitations in relation to which this research can contribute to its improvement.

## SAMR: capabilities and limitations

In this section, we answer the first research question: What are the potentials and limitations of the SAMR model to understand the process of integrating ICT in teaching practices?

The SAMR model is well known among teachers and practitioners of education (Baz et al., 2018; Hilton, 2015; Kimmons & Hall 2018). The model has demonstrated good practical functionality by guiding different uses of ICTs in teaching practices (Lyddon 2019) and an easy set of steps helping teachers in the process of integrating different technologies (Baz et al., 2018). At the same time, there are studies that point to limitations of the model, related to the weak theoretical foundation and the lack of empirical evidence in peer-reviewed studies (Green 2014; Hamilton et al., 2016). In our research, the SAMR model proved to be useful for understanding teachers’ experiences with the uses of ICTs, as well as allowing us to recognize its limitations.

The SAMR model was developed by Puentedura (2006, 2014, 2020), when the author analyzed the uses of ICTs by teachers in elementary and secondary education. Each initial of the acronym SAMR represents a hierarchical formative level of ICTs integration: Substitution, Augmentation, Modification and Redefinition. Based on this four-level taxonomy, teachers are expected to be able to describe and categorize the uses they make of ICTs. This comprehensive character of the model also allows managers and researchers to assess the process of incorporating technologies, in the institutional and scientific scope. In the first two levels (Substitution and Augmentation), technologies are used in the range of improving teaching performance, to improve learning through the uses of technologies. The last two levels (Modification and Redefinition) refer to the transformation range, with structural changes in teaching performance through the uses of technologies.

The SAMR model focuses on the specifics of the tasks of teachers who incorporate ICTs aiming at different qualitative gains, classified by each hierarchical level of the model. Under Puentedura’s terms (2014), the teacher’s goal is to build a simple SAMR ladder that can be linked to Bloom's Revised Taxonomy. To that extent, as the task moves from lower to higher levels in the taxonomy, so does movement from lower to higher levels in the SAMR model. In this process, it is essential for the teachers to analyze: (a) whether there have been changes in the way they teach; (b) whether ICTs were used reflexively; (c) whether continuous assessment of the task was performed; and (d) whether it was possible to promote student learning.

The first level-Substitution-only involves changing an analog technology (Hamilton et al., 2016), or digital when it is less efficient (McKnight et al., 2016), by another digital technology, without causing changes in teaching practice. For example: the teachers can replace acetate transparencies and the overhead projector with Power Point because it allows for more agile presentations, easily modifiable in time, more dynamic with hyperlinks to websites, videos, images, etc. Despite the effort dedicated by the teacher to use Power Point technology, all this did not seem enough for him to change the personal meaning attributed to his practice.

The second level-Augmentation-in addition to the substitution of one technology by another, it is possible to observe small-scale improvements, which do not yet imply robust changes in the teacher's practice system. Technology, at this level, enhances the teaching experience by adding features to the process that would not be possible without it, in addition to enabling the deepening of content, learning and favoring student engagement. For example: the teacher can use websites and blogs to present content that is updated or not available in school library materials; or even, the teacher can ask students to research a certain topic on the internet and classify different points of view, and even participate in the comments section of a blog or webinar.

The third level-Modification-implies some transformation of the teacher's practice in relation to the tasks planned. From this level onwards, the school's physical barriers are eliminated, in order to bring it closer to the learning that takes place in different contexts through which students transit. For example, the teachers can create a document in the cloud and engage students in collaborative editing of that material, that can be in different times and spaces. In this task, students can use different applications and forms to research, compile and defend data. It is possible to observe changes in teaching practice when it becomes more flexible and favorable to the expression of students' positions.

At the fourth level-Redefinition-technology takes on the role of redefining teaching practice, allowing for the creation of new tasks, means and pedagogical strategies that would previously be unthinkable or inconceivable without ICTs. Compared to Bloom’s taxonomy, this is the last level of higher thinking, implying that students, for example, elaborate, produce, devise or invent tasks derived from the teacher's task. For example: students are encouraged by the teachers to develop authorial and collaborative texts that can serve as a source of research for other students at the school. This task can result in the creation of social applications, demonstrating the engagement of students with problems of their own community and providing them with new experiences and learning opportunities.

Despite the model’s popularity, it has been repeatedly criticized for its lack of theoretical foundation and empirical evidence. It is important to highlight that Puentedura developed his work related to SAMR in a very unsystematic way, basically from slides, available on his personal website. Furthermore, there are few connections to theory and previous research, and there are qualitative or quantitative empirical limitations, which often lead to representational and application misunderstandings (Green 2014; Hamilton et al., 2016).

Despite criticisms of the model, it is used in different parts of the world (Baz et al., 2018; Blundell et al., 2020; Hilton, 2015; Kihoza et al., 2016; Kimmons & Hall 2018). Like these authors, we appropriated the SAMR model to understand the uses of ICTs in teaching practices. However, we soon realized that the application of the model without any theoretical guidance led to a simple categorization and prescription of the uses of ICTs, rather than recognizing how they helped to achieve the learning objectives and to favor the participation of teachers and students in contexts of specific activities.

Considering the sociocultural perspective, together with the learning parameters supported by a new learning ecology (Barron 2006; Coll 2018a), we base our arguments on the importance of ICTs integration intrinsically related to a theoretical position in which learning and teaching form a contingent dyad. And, in this direction, we propose reflections that can lead to the improvement of the SAMR model.

## The framework of a new learning ecology

In the current scenario, mediated by the massive uses of ICTs, the physical limits that delimited the possibilities of human interactions have been overcome. As a result, the teaching and learning process began to take place in spaces other than the school, challenging, on the one hand, the school (or the university) as the only privileged context of formal learning and, on the other hand, schooling as a linearly and temporally delimited stage, mediated by specific educational actors. In other words, in contemporary times, the teaching and learning process has been extended throughout life, consisting of ubiquitous spaces of interactions between people and machines.

Several authors (Barron 2006; Brown, 2010; Coll, 2018a,b; González-Sanmamed et al., 2019; Sangrá et al.,^2019^) claim that ICTs have helped to enhance and diversify resources, activities and interactions, altering, according to Coll (2018a), a set of important changes in the parameters of human learning: where, with whom, what, for what and how people learn. Such changes were not a consequence of ICT alone, but their ubiquity helped to shape a new ecology.

Thus, the new learning ecology is characterized by a multiplicity of educational scenarios, resources and agents dispersed in time and space, allowing for alternative and varied ways for the teaching and learning process and human development (Coll 2018a; Engel et al., 2018), in what concerns the uses of cultural artifacts such as ICTs.

Recognizing and enhancing this ecology means thinking, planning and executing with educational intentionality more student-centered teaching practices, implying: recognizing emerging learning spaces; integrate students’ interests into school content; facilitate their guidance and follow-up and develop more flexible assessment strategies. In this way, the teacher also helps students to build a frame of reference about how they learn, in what contexts and/or resources they use to provide new learning opportunities (González-Sanmamed et al., 2018).

## Research context

We developed this research in a public institution in the federal education system in Brazil, which serves students from high school to graduate school, including professional and technical courses. The participants are 116 teachers, 52.6% women and 47.4% men, aged between 25 and over 60 years of age, with more than 80% holding a master’s and/or doctorate degree and classroom experience between 3 and 11 years old. It is essential to mention that the teachers worked simultaneously at different levels and modalities of teaching, for example, secondary education plus technical education.

We applied a research instrument (questionnaire) in which teachers declared the development of teaching practices and how technologies added value to them. This questionnaire is part of a doctoral research on the innovation of teaching practices during emergency remote teaching, approved by an ethics committee (< https://plataformabrasil.saude.gov.br/login.jsf > , CAAE number 24697019.4.0000.5540). Before applying the questionnaire to teachers, we validated the instrument with experts and then carried out the pilot study (< https://repositorio.unb.br/handle/10482/43961 >).

Considering that our concern was centered on how teachers carried out the integration of technologies recognizing a new learning ecology, we mapped 16 teaching practices built in this framework (see Table 1). We presented these practices to teachers in the questionnaire and asked them to register whether they were developing them and, if so, to register for each of them the level of contribution of ICTs by reference to the SAMR model. In addition, we also asked teachers to register an experience in which ICTs allowed them to reformulate their teaching practices.

**Table 1:** Teaching practices within the framework of the new learning ecology

Core	nº	Description
Extension	P1	Search and select information related to teaching content
	P2	Offer multiple sources of information search for students
	P3	Set up collaborative workspaces for students (in person or distance)
	P4	Propose interdisciplinary activities that connect contexts and content from different areas
	P5	Encourage students to publish their productions to the community (internal and external)
	P6	Provide feedback to students on their learning outcomes
Personalization	P7	Perform active methodologies (learning based on projects, cases, problems, gamification, etc.)
	P8	Produce activities aimed at the practical experimentation of the studied contents
	P9	Plan activities that consider the needs, interests, and learning goals of the students
	P10	Provide space for students to make decisions about what activities to do or how to do them
	P11	Reflect with students their interests, identifying opportunities and resources to learn
	P12	Help students reflect on how to learn, identifying their strengths and weaknesses as learners
	P13	Help students establish relationships between what is done in the classroom and their reality
	P14	Encourage student involvement in social issues and values
	P15	Monitor the development of students in different learning activities
	P16	Share, with other teachers, information regarding the monitoring of students

Source: research data, 2020

It is important to mention that when the questionnaire was applied, the institution did not present a program, project or formal research on the integration of technologies, which means that the integration was linked to the teacher’s private initiative, forced by the need for remote teaching. In this sense, when analyzing the data, we emphasize the experiences of teachers and the self-reflection associated with the process of integrating technologies into teaching practices.

## Results and discussion

According to our data, we found that the teaching practices with the highest percentages of development had ICTs integrated to the levels of Augmentation and Modification in the hierarchy of the SAMR model. Practices P10 (6.0%), P11 (8.6%), P12 (8.6%), P13 (8.6%) e P14 (12.1%) received the highest percentages for the Substitution level, that is, the ICTs have not contributed significantly to their development. Such practices have at their core the support of teachers to students so that they reflect on their learning interests, relationships and social implications (see Table 2).

**Table 2:** Levels of contribution of ICTs in teaching practices

	%				
	ICTs do not contribute to this practice	ICTs allow augment the scope of this practice	ICTs allow to modify this practice in part	ICTs allow to completely redefine this practice	Total
P1	0.9%	34.5%	42.2%	21.6%	100%
P2	−	38.8%	34.5%	20.7%	100%
P3	1.7%	22.4%	37.1%	18.1%	100%
P4	1.7%	28.4%	30.2%	15.5%	100%
P5	1.7%	23.3%	19.0%	13.8%	100%
P6	3.4%	34.5%	42.2%	14.7%	100%
P7	1.7%	27.6%	29.3%	17.2%	100%
P8	5.2%	31.9%	32.8%	9.5%	100%
P9	3.4%	33.6%	37.9%	12.9%	100%
P10	6.0%	21.6%	21.6%	10.3%	100%
P11	8.6%	29.3%	28.4%	5.2%	100%
P12	8.6%	26.7%	22.4%	8.6%	100%
P13	8.6%	37.9%	31.9%	9.5%	100%
P14	12.1%	29.3%	25.0%	6.0%	100%
P15	5.2%	31.9%	38.8%	13.8%	100%
P16	3.4%	29.3%	27.6%	9.5%	100%

Source: research data, 2020

In turn, practices P1 (21.6%), P2 (20.7%), P3 (18.1%), P4 (15.5%) and P7 (17.2%) were those with the highest percentages at the Redefinition level, that is, they indicate that ICTs made it possible to redefine them significantly. Due to the characteristic of technologies in allowing the digitization of information and integrating different media, it was possible for teachers to extend their sources of research and content selection that were previously dispersed in different physical media. Thus, this characteristic was taken as a redefinition for teachers. In addition, ICTs allowed teachers to rethink the teaching and learning process, going beyond the space–time limits of the institution to the construction of more collaborative virtual spaces for learning and dissemination of student production, as indicated in practices P3, P4 and P7 (see Table 2).

In addition to these data, teachers made a free record of an experience in which their practices were redefined by different uses of ICTs. In this record, they should present contextual information such as: where and when the experience took place; who was involved; which type of teaching; what were the goals; which ICTs were used; and what were the results found. Of the 116 teachers, 66.4% registered their experience.

Teachers declared positive experiences in reference to the improvements achieved from the functionalities presented by ICTs, taking them as a redefinition of their own practice. However, from the SAMR model, which helped us to analyze the teachers' reports, we observed that most of the declared experiences tended to integrate ICTs in the levels of Augmentation and Modification, corresponding, according to the model, to the passage of the range of improvement to transformation.

However, since the interest of our research focused on the experiences of teachers, we cannot disregard what they themselves understand by redefining teaching practice, especially when such experience was recorded in the context of the emergency remote teaching. Besides, for a sociocultural perspective, it is important to understand the teacher as an agent of change, with emphasis on the meanings and intentions of their practices developed in concrete situations.

In this sense, we put in tension the statements of teachers, seeking to identify how the uses of ICTs helped them to develop and improve their teaching practices. Below, we are going to analyze some excerpts from the experiences declared by these teachers and present our reflections and contributions to the SAMR model.

In this sense, we put in tension the statements of teachers, seeking to identify how the uses of ICTs helped them to develop and improve their teaching practices. Below, we are going to analyze some excerpts from the experiences declared by these teachers and present our reflections and contributions to the SAMR model.

- **Substitution Level**
  […] Students needed to listen to the podcast and submit an analysis/review on the content. It was a podcast of about 30 minutes. Many complained that they were unable to access through their cell phones. Those who managed to access informed that they did not have the patience to listen until the end. Few demonstrated to have enjoyed the activity. It was a somewhat frustrating experience. [Teacher D09]
  […] I used the film “Cast Away” to address the sociological elements. I don't have much experience using more modern and interactive technologies. I think video is still an effective technology. [Teacher D60]

At this level, we did not observe changes in teachers’ practices. In both experiences, the teachers only replaced the technologies, as, instead of using the well-known slide to expose the contents, they opted for the use of podcasts and video, respectively. Furthermore, the role of the teacher as a central agent of the educational process and mediator of knowledge, it seems, remained intact.

- **Augmentation Level**
  My experience was in the preparation of teaching material (handouts). I used a graphic construction site, managed to insert case examples through YouTube links, newspaper articles, processes and then posted it on the VLE [Virtual Learning Environment]. In a single material, students had several interactions for learning and accessibility (augmented font, videos with subtitles, etc.). [Teacher D63]
  […] I chose to work on one of the courses via Google Classroom. There wasn't exactly a redefinition of the course's learning objectives, but there was a reorganization of the contents in different formats (such as podcasts and videos, which until then I hadn't explored in face-to-face classes). I managed to schedule the activities week by week in a more organized way than when I did for in-person courses and I realized that it was much simpler for students to follow the activities with the use of technology. [Teacher D20]

At this level, technologies added more value to teaching practices, as they became more accessible, fun, interactive and led to more student engagement. Both teachers expanded the scope of their activities and organized multiple information to meet the learning styles of students. It was also possible to observe that the uses of ICTs demanded efforts from the teachers, but these were converted into positive results. The technologies added new functionalities to practices that would not be possible otherwise, but did not effectively transform them.

- **Modification Level**
  We developed an activity integrated with other disciplines. […]. To do so, students watched videos before class, responded to a guided study and after the synchronous class, responded to a self-correcting questionnaire on the Google platform. The result of the proposed activity was excellent, as in addition to previously consulting the material in video, podcast and texts, they managed to associate many elements even before the synchronous class. The interaction was much greater due to the prior engagement with the topic, by filling out the form and with the materials. The results were better than expected when we prepared the class. [Teacher D08]
  Biology, physics, chemistry and math teachers came together to apply a project methodology to high school students. Each group of students had autonomy to choose their study topic from a portfolio. The groups developed the activity through the use of different ICTs […]. Thus, the advising teacher could monitor the development of the work and suggest corrections and improvements during the process. […]. The degree of integration achieved between the disciplines was much greater than at any time before the adoption of ICTs. [Teacher D10]

At this level, teaching practices were modified with ICTs. Both teachers redesigned activities beyond the confines of the classroom. In the first experiment, the teacher implemented the inverted classroom. In the second experience, the teacher focused on the development of skills and abilities from an interdisciplinary project, whose integration of ICTs allowed to enrich it. In this sense, it was possible to observe changes favorable to a new learning ecology, as the teachers developed active methodologies, allowed more openness to the students' interests and invited them to be co-responsible for the progress of joint activities.

- **Redefinition Level**
  The experience of approaching ICTs came in the planning of Integrators projects […]. Students studied and established ways to create projects that develop the skills and competences of different courses. The result of this integration in the technical course was the “Viver de Eventos” Project. […], in which students developed a cycle of events encompassing the courses of hosting facilities, people management, and leisure and entertainment. This experience helped me to see the possibility of interaction between different contents, skills and competences of the course, this entire process being done through ICTs. [Teacher D07]
  During the pandemic, students on the Proeja (youth and adult education) course produced videos with content in English in a restaurant/bistro. The script was produced by the students under the supervision of the teacher of this technical area (Beverage Harmonization), [...]. The classes took place through Google Meet, the technical area teacher discussed examples of bar and restaurant services through videos, the English teacher and the intern from the university course in English Language developed gamification activities to practice functional dialogues in English. Lexical and discursive activities were used through Quizlet, Kahoot, Collaborative Glossary via Google docs, wordle and infographic produced on canvas […]. For the review of the scripts (script) and oral practice of the dialogues, a variety of activities were carried out through WhatsApp […]. At the beginning of the pandemic, we had students who watched the class on their cell phone, on the street, to have access to the neighbor's internet. At the end of the course, there was a student living on the street, in a tent due to the economic crisis and unemployment […]. The creation of the educational product by the students enabled the integration of the technical area and the propaedeutic areas, the linguistic, digital and critical literacy of the students. [Teacher D47]

At this level, the teaching practices reported would not be possible to develop without ICTs. We can observe, at the level of redefinition, that the teachers went beyond the limits of their own contents, moving from a uni-disciplinary view to a multidisciplinary and transversal view, involving the participation of the external community. The teachers organized the activities by integrative, dynamic and active projects, with the participation of other educational actors. Students' interests were valued, they made decisions about project activities and had their needs adapted. In addition, it was possible to observe a constant evaluation by teachers regarding the achievement of their learning goals, considering the material conditions of existence of students during the pandemic.

## Propositions to the SAMR model

In this section, we will offer contributions to the improvement of the SAMR model based on the literature and our research data. We will answer the remaining research questions. Research question 2: How can the data from this research improve the SAMR model? Research question 3: How can the data from this research help to understand the integration of ICTs in teaching practices with a view to overcoming second-order barriers?

Our proposal follows a path very similar to what did Hamilton et al. (2016). These authors performed a critical review of the model based on three aspects: (1) absence of context in the model; (2) emphasis placed on the technology as a product rather than as a process; and (3) focus on hierarchical and linear structure.

We highlight four propositions to the SAMR model that help to integrate ICT into teaching practices, in a functional, flexible and sensitive way to the context and needs of educational actors: (1) implications of the sociocultural context; (2) possibility of transitioning between the different levels of the SAMR model; and (3) roles and teaching practices within the framework of a new learning ecology. We end this section by presenting a table that can serve as a reference for teachers.

### Implications of the sociocultural context

The SAMR model can lead to the risk of a prescription, or even hasty generalizations about ICT, nullifying factors such as the complexity of the relationship between students and teachers in reference to the context and process of teaching and learning. The integration of technologies depends on several interrelated factors that can be mistakenly disregarded in a purely technical appropriation of the model, leaving teachers susceptible to the consumption of technologies to the detriment of pedagogical value.

In the process of ICTs integration, the historical, institutional and cultural events, must necessarily be considered. For example, during remote teaching, the quality of teachers' experiences was affected as well as their relationship with technologies. Depending on the technologies they had available and the conditions of students' access to these technologies, the teachers' performance had to be shaped or adjusted (Lennox et al., 2021; Selvaraj et al., 2021; Seufert et al., 2021).

A decontextualized reading of the SAMR model could lead to prescriptive uses of ICTs, only at the Substitution or Augmentation level, during the transposition of face-to-face activities to remote teaching. However, it was not the use of ICTs that was the most important aspect, but the opening of teachers to the heterogeneous needs of students. In general, the pandemic context required teachers to rethink their practices, making them more sensitive to the specific needs of the moment. And the uses of ICTs were conditioned to these needs.

### Possibility of transitioning between the different levels of the SAMR model

The SAMR model conveys the idea of a linear and progressive evolution between hierarchical levels, as if the best results or performances were concentrated in the highest levels of the model. In this logic, the teachers goes from a lower to a higher level without the possibility of transitioning or returning to lower levels, depending on contextual factors or events and new learning objectives. For example, teachers who participated in the survey of Hilton (2015) distributed the SAMR levels throughout the school year considering the practical setting from an instructional objective. According to the authors, teachers strived to reach higher levels of the model, but they did not neglect the base levels. In other words, there was an attempt by teachers to redefine the practice, but this redefinition was sustained and complemented by other levels.

Most teachers who participated in the survey of Geer et al. (2015) demonstrated to be in the SAMR model's enhancement range, that is, in the Substitution and Augmentation levels, although some moved towards the transformation range. As in Flanagan (2016), the authors of the first study showed difficulties in the correlation between the uses of ICTs and the different levels, which could be a limitation of the model itself, but also an indication that teachers did not necessarily go through the four levels sequentially. McKnight et al. (2016) also reported that teachers tended to overlap the uses of ICTs within the SAMR levels, especially when there are layers of virtual and face-to-face learning contexts and distinct tasks where ICTs add different values.

In this way, we understand that it is problematic to deal with a process that is complex and dynamic in a linear way. It is possible for teachers to move between different uses of ICTs, linking these uses to the context of application of their practices, their intentions, objectives and learning outcomes. So, for example, it is possible that a teacher with good command of technologies can make use of some device at the Substitution level because the teacher considered it to be the best strategy for a given time and objective. What is important is that there is clarity in this definition, confirmed by the pedagogical intentionality and (self) evaluation of the teacher, reflecting the dynamic, continuous and fluid nature of the teaching and learning process.

In this way, the uses of ICTs are defined and transformed within the joint activities between teachers, students and contents, which form an interactive triangle (Coll 2014; Coll et al., 2008a). Thinking about this triangle and the layers of contexts and activities in which teachers and students are involved, it is important to understand how ICTs can help them to build knowledge and, especially with regard to teachers, offer the necessary scaffolding for students' learning.

For example, a teacher can use the Virtual Learning Environment (VLE), at the Substitution level of the SAMR model, to make teaching materials available instead of making them available in a physical space. In addition, one can use Google’s various resources to develop, for example, P3 (Set up collaborative workspaces for students) and P9 (Plan activities that consider the needs, interests and learning goals of students) teaching practices, compatible with Modification and Redefinition levels. This means that in a relationship between the macro and micro dimensions of the teaching and learning process, there are multiple possibilities for teaching activities.

Therefore, looking at the model in motion helps to minimize the focus on technology, which ends up being one of the main criticisms attributed to the SAMR model. We believe that when technologies are consciously integrated into teaching practices, there are changes that overcome second-order barriers and improve the teaching and learning process, consequently, the results of this integration feed more varied and creative uses of ICTs. Thus, the mediation potential of these technologies as cultural artifacts allows the planning, regulation and guidance of the activities developed, introducing important changes.

### Roles and teaching practices within the framework of a new learning ecology

Based on our data, we observe that reports of transformations in teaching practices are associated with changes in the roles of teachers and students in the classroom, which is in line with the results achieved in the studies of Blundell et al. (2020) and McKnight et al. (2016). Considering the self-reports of the teachers participating in our research, the transforming uses of technologies focused on active methodologies, such as the inverted classroom, problem-based learning and the development of integrative projects. These are student-centered methodologies, which demanded more agency, responsibility, engagement and autonomy from them. In turn, teachers began to assume a mediating role, as co-facilitators of learning experiences.

These significant role shifts challenge traditional pedagogies and practices. For Blundell et al., (2020), more student-centered pedagogies involve changes in teachers' frame of reference, their beliefs, attitudes and habits, favoring greater student engagement and classroom innovation. Likewise, for Prestridge (2017), Tondeur et al., (2016), and Vongkulluksna et al., (2018), teachers who develop active methodologies, hold constructivist beliefs and practices, and the technologies are used to improve the curriculum. On the other hand, teachers who develop practices centered on their own potential to transmit knowledge tend not to perceive technology as essential for the teaching and learning process (Tondeur, et al., 2016).

The value of ICTs is not something intrinsic to them, their potential is something built based on what is done with them by the teacher, when the teacher manages to improve, by adding value to the teaching and learning processes. For example, we can cite the classic situation in which a teacher divides students into groups and puts them to search for information on the internet, resulting in behavior such as ‘Googling’, a term used by Prestridge (2017) to refer to the mistaken perception that making students look for information on the internet means that they are learning, evaluating, reflecting and adopting a critical and collaborative perspective. In this case, in fact, what was the added value of ICTs to the teaching and learning process?

In this sense, to guide the pedagogical uses of ICTs, it is important to be clear that learning is about a process of construction and attribution of meaning to content, and teaching, a process of systematic, sustained and adjusted help to the construction of meanings. The teaching–learning dyad exists and comes to life thanks to the interactions in the joint activity while teachers and student work the contents and tasks over time, being able to use ICTs as important cultural artifacts.

In view of the above reflections and recognizing the value and practicality of a visual model, we present the table below (see Table 3) in order to help teachers analyze how ICTs can add different values to practices, always linking them to the context and its conditions of mediation. Therefore, it is important to say that, when analyzing the pedagogical uses of ICTs, we must place them within the scope of the joint activity in which teachers, students and the contents that are the object of the teaching and learning process are involved.

**Table 3:** Integration of ICTs

Context of ICTs mediation			
	Substitution	Augmentation/Modification	Redefinition
SAMR levels	Teaching practices that can be developed without ICTs	Functional and fun teaching practices with the uses of ICTs	Teaching practices impossible to be developed without ICTs
Tendency of each level	Keeping learning results despite the insertion of new technologies	Enriching learning experiences without bringing significant changes to learning outcomes	Significantly transform learning outcomes
Teaching intention (examples)	- Increase communication, interaction and productivity. - Decrease student and teacher effort	- Expand the reach of teaching materials, classes, content, tasks, etc. - Integrate and interconnect contexts, resources, information and people.	- Develop multidisciplinary and transversal activities. - Collaborate with the construction of networked content (autonomy and authorship).
ICTs use strategies (examples)	- Produce slides instead of the teacher writing on the blackboard. - Provide multiple choice questions for tests/exams in learning platform instead of using paper. - Upload teaching materials to other sites/devices.	- Produce educational materials with hyperlinks and with accessibility. - Do research, share information or get in touch with social actors on the network. ICTs mediation. - Create resources and virtual learning environments in addition to the classroom.	- Use 3D simulation platforms (theory and practice integration). - Produce content with students in collaboration sharing information with teachers. - Produce integrative projects with social engagement of students
Teacher’s reflection (examples)	Has the insertion of the new technology favored communication and interaction? Has the insertion of the new technology favored the productivity of students and teachers?	Has technology enhanced the students' learning experience, reducing the distance between the school and their reality? Have students started to feel more engaged and recognized as learners?	Has technology favored student collaboration and autonomy? Has technology significantly improved student learning outcomes?
Added value	Conservative	Enriching	Transformative

Source: by the authors (adapted from the SAMR model)

When we look at the SAMR model, we notice that teachers used ICTs more at the Augmentation and Modification levels, corresponding to the passage from the enhancement to the transformation range. Precisely in this passage lay the difficulty in analyzing the uses of ICTs according to the model's guidelines. Thus, we propose to consider both levels together, in order to clarify the tendency of each one of them, considering the intentions, objectives and strategies of the teacher, which can even be modified during the joint activity.

In this sense, the table allows both horizontal and vertical reading as it recognizes the fluid nature of the act of teaching and learning, making it possible for teachers to move between the different levels of the model as it reflects on the gains favored by the mediation of ICTs: conservative, enriching or transformative.

In view of these multiple layers of ICTs mediation, there is simultaneity in the uses of ICT, which add different value as it affects the interrelationships of the elements of the interactive triangle and, consequently, the teaching and learning process. Understanding this is important for us to understand how teachers produce their experiences from the mediation of these important cultural artifacts, helping to overcome second-order barriers.

## Conclusion

We analyzed teachers' experiences on the uses of ICT, in how they integrated these technologies to develop teaching practices. Based on the data, we propose a more flexible, non-hierarchical understanding of the SAMR model, based on sociocultural theory and on the parameters of the new learning ecology. Each level of the model does not need to be understood as better or worse or refer to a ladder on which teachers advance to higher levels. We assess that the contextual and flexible nature of the model can result in more focus on pedagogical intentionality.

We hope that our efforts can help to improve the SAMR model and to understand the relationship between teachers and the uses of ICT, as well as helping to overcome second-order barriers that prevent effective uses of these technologies in the teaching and learning process.

## Data Availability

The datasets used and analysed during the current study are available from the corresponding author on reasonable request. Please contact author for data requests.

## References

[CR1] Backfisch I, Lachner A, Stürmer K, Scheiter K (2021). Variability of teachers’ technology integration in the classroom: A matter of utility!. Computers & Education.

[CR2] Barron B (2006). Interest and self-sustained learning as catalysts of development: A learning ecologies perspective. Human Development.

[CR3] Baz EH, Balçıkanlı C, Cephe PT (2018). Introducing an innovative technology integration model: Echoes from EFL pre-service teachers. Education and Information Technologies.

[CR4] Blundell C, Lee K, Nykvist S (2020). Moving beyond enhancing pedagogies with digital technologies: Frames of reference, habits of mind and transformative learning. Journal of Research on Technology in Education.

[CR5] Brown JS (2010). Growing up digital: How the web changes work, education, and the way people learn. Change.

[CR6] Coll C, Mauri T, Onrubia J, Coll C, Monereo C (2008). La incorporación de las TIC a la educación: del diseño tecno–pedagógico a las prácticas de uso. Psicología de la educación virtual Enseñar y aprender con las tecnologías de la información y la comunicación.

[CR7] Coll, C., Onrubia, J., & Mauri, T. (2008b). El análisis de los usos reales de las TIC en contextos educativos formales: una aproximación socio-cultural. *Revista Electrónica de Investigación Educativa*, *10*(1), 1–18. https://www.scielo.org.mx/pdf/redie/v10n1/v10n1a1.pdf

[CR8] Coll, C., & Martí, E. (2014). La educación escolar ante las nuevas tecnologías de la información y de la comunicación. In C. Coll, J. Palacios, & A. Marchesi (Orgs.), *Desarrollo psicológico y educación. Psicologia de la educación* (pp. 623–648). Alianza.

[CR9] Coll, C. (2018a). Personalizing school learning, a requirement of the new learning ecology. In C. Coll (Coord.), *Personalización del aprendizaje*. (pp. 5–11). Graó. http://psyed.edu.es/archivos/grintie/Coll_2018a_PersonalizingSchoolLearning.pdf

[CR10] Coll, C. (2018b). Learning processes that generate learning with personal value and strategies for personalizing school learning. In C. Coll (Coord.), *Personalización del aprendizaje* (pp. 14–18). Graó. http://psyed.edu.es/archivos/grintie/Coll_2018b_LearningProcessesThatGenerateMeaning.pdf

[CR11] Engel, A., Coll, C., Membrive, A., & Oller, J. (2018). Information and communication technologies and students’ out of school learning experiences. *Digital Education Review*, *33*, 130–149. https://files.eric.ed.gov/fulltext/EJ1183663.pdf

[CR12] Engel A, Coll C (2022). Entornos híbridos de enseñanza y aprendizaje para promover la personalización del aprendizaje. RIED.

[CR13] Flanagan, M. (2016). *The effects of a one-to-one iPad initiative: a case study* (Doctoral Thesis). The University of Wisconsin-Milwaukee, USA. https://dc.uwm.edu/etd/1365/

[CR14] Geer R, White B, Zeegers Y, Au W, Barnes A (2015). Emerging pedagogies for the use of iPads in schools. British Journal of Educational Technology.

[CR15] González-Sanmamed M, Muñoz-Carril PC, Santos-Caamaño FJ (2019). Key components of learning ecologies: A delphi assessment. British Journal of Educational Technology.

[CR16] González-Sanmamed M, Sangrá A, Souto-Seijo A, Blanco IE (2018). Ecologías de Aprendizaje en la era digital: desafíos para la educación superior. Publicaciones.

[CR17] Green, L. S. (2014). Through the looking glass. *Knowledge Quest, 43*(1), 36–43. https://www.learntechlib.org/p/157541/

[CR18] Hamilton E, Rosenberg JM, Akcaoglu M (2016). The substitution augmentation modification redefinition (SAMR) model: A critical review and suggestions for its use. TechTrends.

[CR19] Hilton JT (2015). A case study of the application of samr and tpack for reflection on technology integration into two social studies classrooms. The Social Studies.

[CR20] Kihoza, P., Zlotnikova, I., Bada, J., & Kalegele, K. (2016). Classroom ICT integration in Tanzania: Opportunities and challenges from the perspectives of TPACK and SAMR models. *International Journal of Education and Development using ICT*, *12*(1). https://www.learntechlib.org/p/173436/

[CR21] Kimmons R, Hall C (2018). How useful are our models? pre-service and practicing teacher evaluations of technology integration models. TechTrends.

[CR22] Law N, Yuen A, Fox R (2011). Educacional Innovations Beyond Technology: Nurturing Leadership and Establishing Learning Organizations.

[CR23] Lennox J, Reuge N, Benavides F (2021). UNICEF’s lessons learned from the education response to the COVID-19 crisis and reflections on the implications for education policy. International Journal of Educational Development.

[CR24] Lyddon PA (2019). A reflective approach to digital technology implementation in language teaching: expanding pedagogical capacity by rethinking substitution, augmentation, modification, and redefinition. Tesl Canada Jornal.

[CR25] McKnight K, O'Malley K, Ruzic R, Horsley MK, Franey J, Bassett K (2016). Teaching in a Digital Age: How Educators Use Technology to Improve Student Learning. JRTE.

[CR26] Prestridge S (2017). Examining the shaping of teachers’ pedagogical orientation for the use of technology. Technology, Pedagogy and Education.

[CR27] Puentedura, R. (2006). *Transformation, technology and education*. http://hippasus.com/resources/tte/

[CR28] Puentedura, R. (2014). *SAMR and Bloom´s Taxonomy: assembling the puzzle.* https://www.commonsense.org/education/articles/samr-and-blooms-taxonomy-assembling-the-puzzle

[CR29] Puentedura, R. (2020). *SAMR, Observation, Analysis, and Action*. http://hippasus.com/blog/

[CR30] Sangrá A, Raffaghelli EJ, Guitert-Catasús M (2019). Learning ecologies through a lens: Ontological, methodological and applicative issues. A systematic review of the literature. British Journal of Educational Technology..

[CR31] Selvaraj A, Vishnub R, Nithin KA, Benson N, Mathew AJ (2021). Effect of pandemic based online education on teaching and learning system. International Journal of Educational Development.

[CR32] Seufert S, Guggemos J, Sailer M (2021). Technology-related knowledge, skills, and attitudes of pre- and in-service teachers: The current situation and emerging trends. Computers in Human Behavior.

[CR33] Tondeur J, Braak J, Ertmer P, Ottenbreit-Leftwich A (2016). Understanding the relationship between teachers’ pedagogical beliefs and technology use in education: A systematic review of qualitative evidence. Education Tech Research.

[CR34] Vongkulluksna VW, Xie K, Bowmana MA (2018). The role of value on teachers' internalization of external barriers and externalization of personal beliefs for classroom technology integration. Computers & Education.

